# When Did HIV Incidence Peak in Harare, Zimbabwe? Back-Calculation from Mortality Statistics

**DOI:** 10.1371/journal.pone.0001711

**Published:** 2008-03-05

**Authors:** Ben Lopman, Simon Gregson

**Affiliations:** 1 Department of Infectious Disease Epidemiology, Imperial College London, London, United Kingdom; 2 Biomedical Research and Training Institute, Harare, Zimbabwe; Instituto de Pesquisa Clinica Evandro Chagas, FIOCRUZ, Brazil

## Abstract

HIV prevalence has recently begun to decline in Zimbabwe, a result of both high levels of AIDS mortality and a reduction in incident infections. An important component in understanding the dynamics in HIV prevalence is knowledge of past trends in incidence, such as when incidence peaked and at what level. However, empirical measurements of incidence over an extended time period are not available from Zimbabwe or elsewhere in sub-Saharan Africa. Using mortality data, we use a back-calculation technique to reconstruct historic trends in incidence. From AIDS mortality data, extracted from death registration in Harare, together with an estimate of survival post-infection, HIV incidence trends were reconstructed that would give rise to the observed patterns of AIDS mortality. Models were fitted assuming three parametric forms of the incidence curve and under nine different assumptions regarding combinations of trends in non-AIDS mortality and patterns of survival post-infection with HIV. HIV prevalence was forward-projected from the fitted incidence and mortality curves. Models that constrained the incidence pattern to a cubic spline function were flexible and produced well-fitting, realistic patterns of incidence. In models assuming constant levels of non-AIDS mortality, annual incidence peaked between 4 and 5% between 1988 and 1990. Under other assumptions the peak level ranged from 3 to 8% per annum. However, scenarios assuming increasing levels of non-AIDS mortality resulted in implausibly low estimates of peak prevalence (11%), whereas models with decreasing underlying crude mortality could be consistent with the prevalence and mortality data. HIV incidence is most likely to have peaked in Harare between 1988 and 1990, which may have preceded the peak elsewhere in Zimbabwe. This finding, considered alongside the timing and location of HIV prevention activities, will give insight into the decline of HIV prevalence in Zimbabwe.

## Introduction

The recently observed decline in HIV prevalence in Zimbabwe is a result of both high levels of AIDS mortality combined with a reduction in incident infections.[1], [2] An important component in understanding these dynamics in HIV prevalence is knowledge of the historical trend in HIV incidence, including at which level and year incidence peaked. Knowledge of incidence trends combined with data on sexual behaviour change and prevention program implementation will aid in understanding the decline in HIV prevalence. HIV incidence may peak naturally when HIV spreads in a previously uninfected population.[3] The timing and level of the peak will be related to dynamics of spread which may be mediated by behaviour change such as reductions in casual partnerships or increased condom use.[4]

Few longitudinal studies of HIV infection exist from sub-Saharan Africa, so empirical estimates of incidence are scare. For Zimbabwe, most incidence estimates that do exist are not from population-based studies, so may be biased and/or are recent, and therefore likely to be well after the peak in infections occurred.[5]–[7] In the absence of an extended time-series of empirical measurements, other approaches are needed to estimate incidence.

Back-calculation is a method of estimating past infection rates. This method has been applied to reconstruct historic incidence rates using a time-series of AIDS cases and to make short-term incidence projections.[8]–[10] However, in virtually all settings where there is a generalised HIV epidemic, there is no reliable surveillance for new AIDS cases. Therefore, in this paper, we use the technique to estimate past infection rates from AIDS mortality statistics. When estimating HIV incidence from AIDS cases, the basic idea is to use AIDS incidence together with an estimate of the incubation time to estimate HIV incidence. Analogously, we use AIDS mortality together with an estimate of survival post-infection to reconstruct the trends in HIV incidence that would give rise to observed patterns of AIDS mortality.

## Methods

The relationship between expected AIDS mortality by calendar time *t, A(t)*, the incident cases *G(s)* at calendar time *s* and the incubation period distribution *F(t)*, is given by the convolution equation:
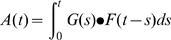
(1)


For most previous applications of back-calculation (see for example Isham [9], [10]) knowledge of *A(t)* comes from AIDS registries collected by public health surveillance. Here we use death registration data as described below. *F(t)* for the current application comes from epidemiological studies that estimated the distribution of survival-post infection. After the incidence curve was fitted, we forward projected the prevalence curve that it would produce. Since more data are available on prevalence than incidence, prevalence estimates were used to validate the models. The procedure for this is described in more detail below.

### Mortality

**Figure 1:**
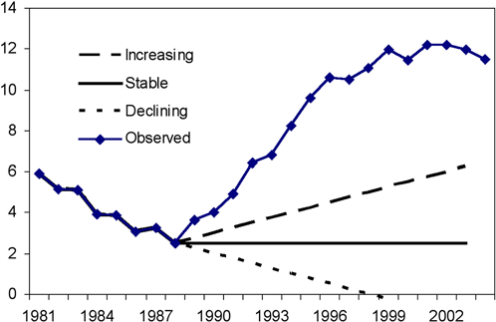
Crude mortality rate from death registration in Harare, with three scenarios of stable, decreasing and increasing underlying non-AIDS mortality. Y-axis is deaths per 1000 population per year.

We estimated AIDS mortality from vital registration data for Harare. Limited data were also available for Bulawayo, and were analysed for comparison. AIDS mortality was assumed to be the excess of deaths that began to occur in the late 1980s. Prior to this upturn, mortality rates were decreasing. We assumed no AIDS deaths prior to the upturn. The year with lowest mortality since 1980 is represented by *CDR(b).* AIDS deaths in year *t, A(t),* were estimated from the difference between the overall CDR(t) and *CDR(b)* and *P:(t)* is the whole population in year *t*.[11] Three scenarios were analysed with regards to non-AIDS mortality: stable background mortality, increasing background mortality and decreasing background mortality (Figure 1). In the stable background scenario:
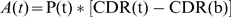
(2)


In the increasing and decreasing scenarios, it was assumed that CDR was changing by 10% per annum, relative to the year of lowest mortality. Where background mortality is decreasing:
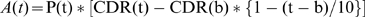
(3)and where background mortality is increasing:
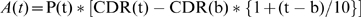
(4)


These estimates of AIDS deaths, based on excess deaths at all ages, will include deaths under age 15, caused by excess mortality due to infections in children, and deaths at ages 60 and over, a few of which may have occurred as a result of infections at ages 60+. However, in order to compare our estimates of incidence and prevalence to more widely available data for the age range 15–59, we assume that deaths outside the age range 15–59 are a negligible fraction of the total excess due to AIDS. Defining N(t) as the number of individuals aged 15–59, we use the following definitions of incidence and prevalence:

Incidence and prevalence

Incidence in the adult population was calculated as the number of incident infections in year *t, G(t),* divided by the susceptible adult population as follows:
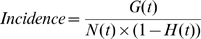
(5)where *N(t)* is the population aged 15 to 59 in year *t*. Therefore, we assumed that all incident cases occurred in people in this age range. It is also assumed that every HIV infection results in an AIDS death, i.e. that there is no other-cause mortality in HIV infected individuals. Mortality rates are typically 10–15 times higher in HIV infected people, so this assumption may result in a small underestimate of the true incidence This incidence is a risk measure, which will closely approximate the rate since the number of annual incident cases is small relative to the whole population size.

Prevalence is calculated using N(t) (15 to 59 year olds) as the denominator, so it is assumed that all infected people fall within this age range. Deaths occurring in the elderly from HIV and deaths resulting from vertical transmission to children have been absorbed in the adult prevalence measure. HIV prevalence in the adult population, *H(t),* was projected after the incidence curves were fitted as follows:
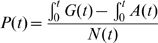
(6)


### Survival post infection

Survival post-infection was assumed to follow a Weibull distribution. Three scenarios were analysed in which the shape (κ) and scale (λ) parameters were derived from three different epidemiological studies of survival post infection. The ‘short’ survival was derived from Ugandan subjects[12], ‘medium’ survival from Western subjects[13] and the ‘long’ survival from the a collaborative study sites (mainly from sub-Saharan Africa).[14] The shape and scale of these three curves differed, therefore generating a range of incidence curves *G(s)*(Appendix S1).

### Modelling the infection curve

Two methods of deconvolution were examined for determining the historical incidence of HIV infection, *G(s):*

#### Statistical deconvolution

AIDS deaths were modelled using a maximum-likelihood regression approach [8] [8] and observed mortality in a given year was assumed to follow a Poisson distribution. Parameters, whose coefficients represented incident HIV infections in a given year, were fitted using generalised linear regression models. The discretised Weibull distribution played the role of the covariates. However, this approach may be “ill-posed” [15] since we are attempting to fit *n* parameters (yearly incidence) to *n* data points (observed deaths). One solution is to smooth the incidence estimates, by assuming incidence changes over longer periods (2 years, for example). However, preliminary analysis using regression approaches (the glm commands in STATA 9.0) revealed that even by smoothing in 5 five year intervals, coefficients were ‘saw-toothed’ and sometimes negative. Therefore, it was concluded that methods that constrain the pattern of incidence were necessary.

#### Functional forms and distributions for HIV incidence

Three separate models were fitted assuming different functional forms of the incidence curve *G(s)*. The log-logistic, gamma, and cubic spline functions were selected because they can represent a pattern of increasing incidence, followed by a decrease and subsequent levelling off. The parameters were fitted by least squares of the residuals between the expected mortality generated by the incidence curve at time *t* and the observed mortality. Both parameters of the log-logistic and the gamma distributions were fitted. For the cubic spline, the following constraints were imposed: the start of the epidemic was assumed to be 7 years prior to the peak, the knot (spline point) was assumed to occur at the peak. Therefore 3 parameters representing the mode (peak year) and maximum (peak incidence) and end year of incidence were fitted. For the cubic spline, which is more flexible, we also fit the incidence curve to empirical measurements of incidence. The incidence and mortality data points were equally weighted. The sensitivity of the estimated peak year and peak level to the assumption that the epidemic began 7 years prior to the peak is presented below.

#### Estimation of confidence intervals

We estimated the 95% confidence intervals of parameters (peak incidence level and peak year of incidence) using the goodness of fit statistics. An *F* ratio was calculated to determine to what level the goodness of fit would be reduced such that the reduced-fit model (p < 0.05) was significantly worse than the best fit model, as described by Motulsky and Christopulous.[16] However, the models produced very close fits to the data. Thus, the confidence intervals were very small (e.g. upper and lower limits of estimates of incidence were equal to four decimal places) and are not presented.

## Results

Crude mortality reached its lowest point in 1988 at 2.6 per 1000 population. We assume no AIDS mortality up until this point and AIDS mortality in the stable background mortality scenario *t* to be CDR(t) - 2.6. In the increasing mortality scenario, the CDR reached levels similar to the early 1980's by the end of the observation period in 2004. In the decreasing background mortality scenario CDR reached 0 in 1998, and was assumed to be zero in subsequent years (Figure 1).

**Figure 2:**
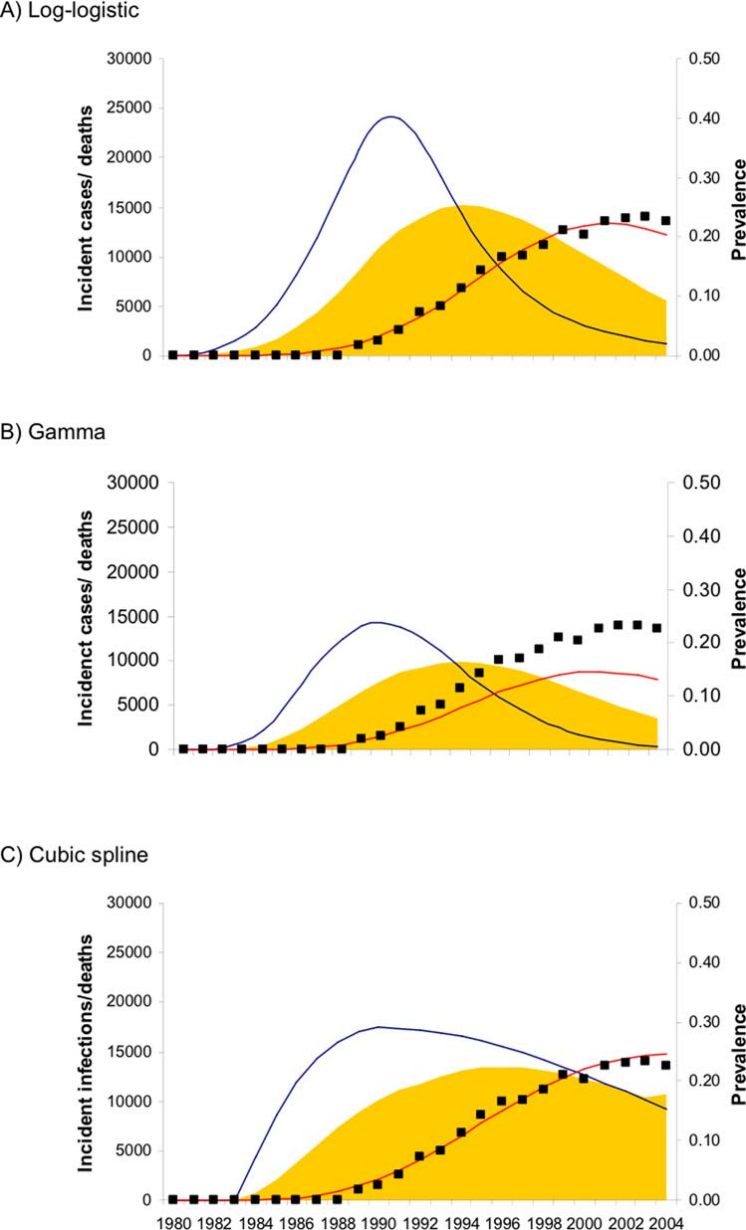
Comparison of three parametric forms of the incidence curve: log-logistic (A) gamma (B) and cubic spline (C). Incidence curves (blue line) and generated mortality curve (red line) was fitted to observed excess AIDS mortality (black points) and prevalence (orange shade). All assume median survival post infection is 11.3 years and stable non-AIDS mortality.

Models assuming a gamma-distributed incidence function failed to generate a well-fitting mortality function (Figure 2B and Table 1). The log-logistic and cublic spline models fitted the mortality data well producing estimated peak incidence rates of 5% to 12% and 4% to 5% per annum, respectively, assuming stable baseline mortality. Log-logistic models tended to produce higher peak levels and later peaks (around 1990/91) compared to the cublic spline. In cubic spline models, the incidence curve was smoother and peaked somewhat earlier (around 1988/1990). These two models were also flexible to different assumptions of background mortality. In the declining background mortality scenario, higher and more recent peaks resulted, which were plausible except that the log-logistic long-survival model predicted a non-credible peak incidence of 20%. The increasing background mortality scenario resulted in implausibly low prevalence, peaking at around 11% and falling to 3% to 5% in 2004. This suggests that background crude mortality levels have not been increasing in Harare death registration.

**Table 1:** Peak rate, year and goodness of fit under different parametric forms of incidence, background mortality and survival-post infection.

Model	Background mortality	Post infection survival	Peak Rate^a^	Peak Year	Goodness of fit
Log-logistic	Decreasing	Min	0.08	1993	0.000626
		Mid	0.10	1991	0.000491
		Max	0.20	1992	0.001011
	Stable	Min	0.05	1992	0.000370
		Mid	0.06	1991	0.000306
		Max	0.12	1991	0.000541
	Increasing	Min	0.03	1991	0.000313
		Mid	0.04	1990	0.000363
		Max	0.08	1991	0.000229
Gamma	Decreasing	Min	0.03	1988	0.009933
		Mid	0.03	1988	0.009917
		Max	0.04	1987	0.014146
	Stable	Min	0.04	1988	0.015465
		Mid	0.04	1988	0.013988
		Max	0.05	1987	0.020026
	Increasing	Min	0.02	1988	0.008181
		Mid	0.02	1988	0.009149
		Max	0.02	1988	0.013801
Cubic spline	Decreasing	Min	0.04	1989	0.000640
		Mid	0.03	1988	0.000592
		Max	0.03	1989	0.000511
	Stable	Min	0.05	1989	0.000307
		Mid	0.04	1988	0.000237
		Max	0.04	1990	0.000181
	Increasing	Min	0.06	1988	0.000724
		Mid	0.06	1988	0.000295
		Max	0.05	1989	0.000189

Varying the assumption in the cubic spline model that the epidemic began 7 years before the peak in incidence resulted in changes in peak year, but not in peak level of incidence. Changing the assumption by 2 years (to 5 or 9) resulted in a less than two year change in the estimate of peak year. For example, in the long-survival stable mortality scenario, peak incidence assuming a 5, 7, and 9 year lag, ranged from 1990.2, 1991.6 and 1992.9, respectively, while the estimates of peak incidence all fell within 4.2 to 4.4%. The fitted end year of incidence fell between 2009 and 2019. Fitting this parameter gave a smoother incidence curve that better represented incidence estimates available from later in the epidemic.

**Figure 3:**
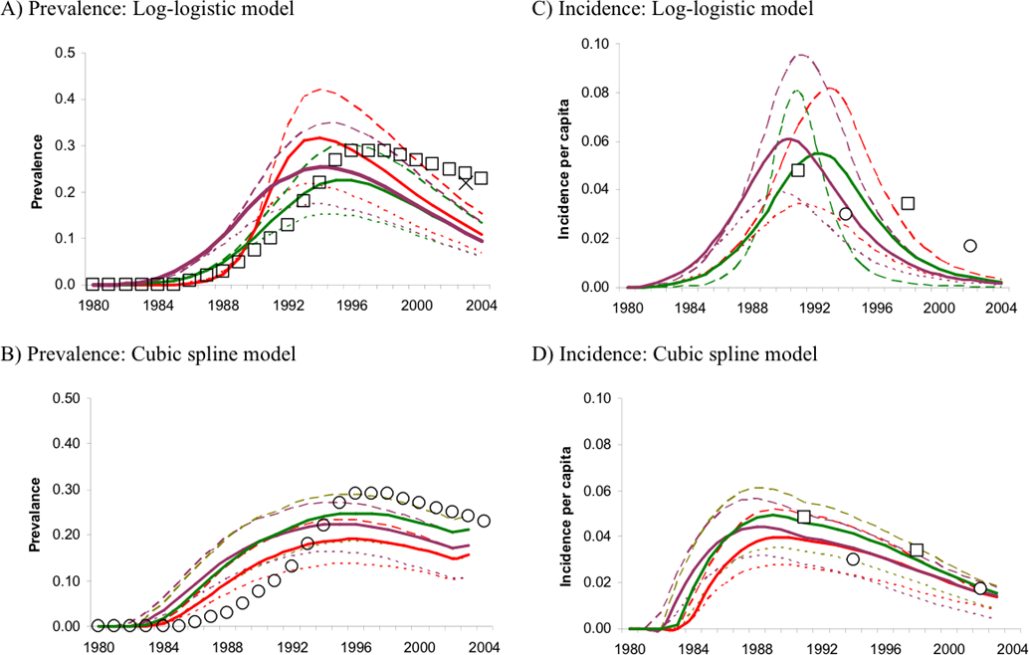
Time trends of adult HIV prevalence (A&B) projected from parameterised incidence curves (C&D). Higher prevalence results assuming declining background mortality (orange lines) compared with stable (green lines) or increasing background mortality (blue lines). Darker shaded lines represent longer survival post-infection; lighter lines represent shorter survival. UNAIDS prevalence estimates are shown for comparison (symbol, ref). HIV incidence measured in Harare male factory workers (○[7]) and Harare post-natal women (□[27]) are plotted alongside modelled incidence in C&D. Prevalence and incidence estimates from back-calculation are for 15 to 59 year olds, empirical and national estimates generally refer to 15 to 49 year olds.

By forward projecting incidence to prevalence, we can compare predicted estimates with national estimates of HIV prevalence, which are based on antenatal clinic surveillance.[17], [18] National estimates of HIV prevalence fit more closely to models assuming declining background mortality, and show a gentler peak more similar to the cubic spline model. If the epidemic progressed faster in Harare than other areas of the country, prevalence may be lower than national levels later in the epidemic (reflected in Figures 3A and 3B). Empirical and national estimates plotted in figure 3 are for 15 to 49 year age groups. Our back-calculation estimates are for 15 to 59 year olds. Therefore, our estimates which will tend to be lower since infections are less common in older age groups.[19]

A shorter reliable time series of death registration is available from Zimbabwe's second largest city Bulawayo.[1] Fitting the cubic spline models, assuming constant underlying mortality, resulted in incidence peaks slightly later (1992 to 1994) and slightly higher (5% to 6%) than in Harare. In Bulawayo, we estimate that prevalence peaked between 16% and 22%, somewhat lower than in Harare (19–26%).

## Discussion

Using cubic spline models, we back-predicted a peak HIV incidence in Harare between 3% and 6%, which occurred between 1988 and 1990. Somewhat less flexible log-logistic models produced later and generally higher peaks. Of the three parametric models tested, we believe that the cubic spline model gives the most plausible back-projection. It may be surprising that the peak in incidence occurred so long ago, but the peak in incidence will precede the peak prevalence by a number of years. Furthermore, it is likely that the epidemic first spread in the capital Harare, then disseminating to other urban areas and rural areas. This theory is consistent with the finding of a later peak occurring in Bulawayo compared with Harare.

This is a novel application of mortality data to reconstruct historical incidence. But the use of crude mortality statistics presents a number of difficulties. For example, urban populations are not closed. Importantly, individuals who become infected in cities may migrate from urban areas to receive care from relatives and ultimately die in rural areas.[20] This would result in an underestimate of mortality and, in turn, incidence and may result in a shift in the peak year towards the past if such migration is increasing. Graveyards in Harare were recently declared “full” [21] so this may in fact begin to occur, if it has not already. On the other hand, people may come to the city to receive medical care, resulting in the opposite bias of increased peak incidence and a more recent peak. This would be especially true if anti-retroviral therapy was widely available in Harare; but this was not the case during the study period. We are aware of no clear evidence of HIV-related migration to suggest one scenario over another.

Reporting delays would result in underestimation of recent mortality; thus, levelling-off of rates may just be an effect of reporting lag. To investigate the sensitivity of the results to reporting delays, we fitted the curves to a truncated series of mortality, using data only up to 2000. The results were not substantially affected. For example, using the medium survival assumption with a log-logistic model predicted a peak incidence of 6% cases per 1000 person years in 1991, essentially the same as when using the full series of data. However, there remains a large degree of uncertainly in the recent estimates of incidence because few of the individuals infected recently would be expected to have died by the end of the survey period. Further, sub-optimal treatment of HIV including monotherapy and treatment of opportunist infection many have affected the survival during the HIV epidemic, but we assume this effect would be small and captured within the range of survival curves.

Our best estimates of peak incidence fall between approximately 4% and 8%. These rates exceed almost all empirical estimates of HIV incidence in general populations, though estimates of incidence are scarce. Furthermore, empirical estimates of incidence are cumulative measures and would have to be performed for a short duration at the right time to have been able to capture peak incidence. Nonetheless, these rates are high and may only occur in areas of high labour migration with a concentration of adults in the peak age of vulnerability.

For Zimbabwe as a whole, completeness of death registration increased, roughly estimated to have risen from 40% and 57% in 1982 for women and men, respectively, to 59 and 85% in 1995, respectively.[22] However, trends and levels of under-registration of mortality in Harare, in particular, are largely unknown. If levels of under-registration in Harare remained constant, reconstructed incidence would be too low, but the timing of the peak would be unchanged. Increasing or decreasing levels of registration, however, would result in biased estimates of both level and timing of incidence trends. Results would be most heavily biased if AIDS deaths were selectively under-registered.

We assumed that all AIDS deaths occurred in adults. In mature epidemics without effective prevention of mother-to-child transmission, there tends to be approximately ten prevalent adult cases of HIV for every infected child under the age of 15.[23] There will be a time lag of prevalence in children from prevalence in adults since child infections are mainly transmitted vertically from mothers. Therefore, the ratio of adult to child cases may be even higher than 10∶1 in a rapidly growing epidemic, which may have occurred in Harare. Combined, this may result in an overestimate of incidence, which may rise to as much as 10% in the later years of the epidemic.

We analysed three scenarios of increasing, decreasing and stable non-AIDS mortality. All three scenarios deserve consideration because different sources of mortality data suggest different trends. For example, DHS estimates of suggest a recent decrease in child mortality [24]. Conversely, it may also be possible that underlying mortality is increasing because of the severe recession of the Zimbabwean economy, degradation of the health services, as well as increase in other causes of death due to the interaction between HIV and diseases such as TB [25] and malaria[26]

Both the cubic spline and log logistic models provide excellent fits to the mortality data. So the confidence intervals, based on goodness of fit statistics, were very narrow. The uncertainty lies more in the choice of parametric form, so the range of results from the different model choices provides an alternative expression in the uncertainty.

HIV incidence is most likely to have peaked in Harare between 1988 and 1990. Given different assumptions about the survival time following infection and different parametric forms of the infection curve, the credible interval in which that incidence may have peaked was between 1988 and 1992. This finding will be used–along with other data on trends in the epidemic-in an ongoing exercise to assess the likely timing of changes in sexual risk behaviour in Zimbabwe and, thereby, to evaluate the importance of different national HIV prevention programmes in reducing rates of infection.

## Supporting Information

Appendix S1 Weibull parameters generating the survival functions
(0.06 MB DOC)
