# Supplementary material for: When Did HIV Incidence Peak in Harare, Zimbabwe? Back-Calculation from Mortality Statistics
Source: PLoS One. 2008 Mar 5;3(3):e1711. doi: 10.1371/journal.pone.0001711 (PMC2248619; doi:10.1371/journal.pone.0001711)
Supplement: Appendix S1 — Weibull parameters generating the survival functions (0.06 MB DOC) [file pone.0001711.s001.doc]

**Appendix S1.** Weibull parameters generating the survival functions.

|  | Weibull parameters | | Mean survival |
| --- | --- | --- | --- |
|  | Scale (λ) | Shape (κ) |  |
| Minimum survival[13] | 2.2 | 11.6 | 9.8 |
| Medium survival [12] | 2.5 | 13.1 | 11.3 |
| Maximum survival[14] | 2.0 | 14.1 | 11.7 |
